# Compatibility of Phase Change Materials and Metals: Experimental Evaluation Based on the Corrosion Rate

**DOI:** 10.3390/molecules25122823

**Published:** 2020-06-18

**Authors:** Milan Ostrý, Sylva Bantová, Karel Struhala

**Affiliations:** Brno University of Technology, Faculty of Civil Engineering, 602 00 Brno, Czech Republic; bantova.s@fce.vutbr.cz (S.B.); struhala.k@fce.vutbr.cz (K.S.)

**Keywords:** phase change materials (PCMs), metals, container, latent heat storage, corrosion

## Abstract

The construction and maintenance of building stock is responsible for approximately 36% of all CO_2_ emissions in the European Union. One of the possibilities of how to achieve high energy-efficient and decarbonized building stock is the integration of renewable energy sources (RES) in building energy systems that contain efficient energy storage capacity. Phase Change Materials (PCMs) are latent heat storage media with a high potential of integration in building structures and technical systems. Their solid-liquid transition is specifically utilized for thermal energy storage in building applications. The typically quite old example is the use of ice that serves as long-term storage of cold. Large pieces of ice cut in winter were stored in heavily insulated spaces and prepared for cooling of food or beverages in summer. In the contemporary use of the principle, the PCMs for building applications and tested in this study must have a melting range close to the desired temperature in the occupied rooms. As the PCMs need to be encapsulated, several types of metal containers have been developed and tested for their thermal conductivity and resistance to mechanical damage, which enhances the performance of these so-called latent heat thermal energy storage (LHTES) systems. Long-term compatibility of metals with PCMs depends, i.e., on the elimination of an undesirable interaction between the metal and the specific PCM. Heat storage medium must be reliably sealed in a metal container, especially if the LHTES is integrated into systems where PCM leaks can negatively affect human health (e.g., domestic hot water tanks). The aim of this study is to evaluate the interactions between the selected commercially available organic (Linpar 17 and 1820) and inorganic (Rubitherm SP22 and SP25) PCMs and metals widely used for PCM encapsulation (aluminum, brass, carbon steel, and copper). The evaluation is based on the calculation of the corrosion rate (CR), and the gravimetric method is used for the determination of the weight variations of the metal samples. The results show good compatibility for all metals with organic PCMs, which is demonstrated by a mass loss as low as 2.1 mg in case of carbon steel immersed in Linpar 1820 for 12 weeks. The exposure of metals to organic PCMs also did not cause any visual changes on the surface except for darker stains, and tarnishing occurred on the copper samples. More pronounced changes were observed in metal samples immersed in inorganic PCMs. The highest CR values were calculated for carbon steel exposed to inorganic PCM Rubitherm SP25 (up to 13.897 mg·cm^−2^·year^−1^). The conclusion of the study is that aluminum is the most suitable container material for the tested PCMs as it shows the lowest mass loss and minimal visual changes on the surface after prolonged exposure to the selected PCMs.

## 1. Introduction

The European Union is committed to developing a sustainable, competitive, secure, and decarbonized energy system by 2050 [1]. Therefore, one of the key issues is the utilization of renewable energy sources (RES) for heating and cooling of buildings. The use of RES instead of burning of fossil fuels (coal, crude oil, natural gas) for building operations significantly reduces greenhouse emissions, and thus contributes to the decarbonization of the built environment. Energy generation from most of RES is not stable and may depend on meteorological situations, e.g., solar radiation and wind velocity. Therefore, thermal energy storage systems are important to reduce the fluctuation of energy production from RES [2]. Building structures (walls, floors) and building services with water tanks represent thermal energy storage potential that can be employed for the adjustment of energy consumption to provide a flexible energy demand [3]. Three possible techniques that can be employed when the significant increase of thermal energy storage should be integrated into buildings are Sensible Heat Thermal Energy Storage (SHTES), Latent Heat Thermal Energy Storage (LHTES), and Thermochemical Heat Thermal Energy Storage (THTES) [4]. LHTES systems use reversible phase change in the storage medium. Although the heat of fusion predominates, a lot of heat is also stored in the solid and liquid state through SHTES. The major advantage of LHTES over SHTES is a much higher energy storage capacity per unit of volume or mass, leading to lower spatial requirements. Therefore, LHTES has the potential to replace common SHTES systems (often represented by water tanks) in energy storage units due to its higher heat-storage density. Heat storage in almost isothermal conditions is another advantage of LHTES, [5]. These reasons suggest that the application of LHTES could be advantageous, e.g., in solar thermal systems, heating and cooling systems, building structures, thermal regulation textiles (smart textiles), concentrated solar power systems, food services, thermal management of automotive engines or spacecraft thermal control [6].

LHTES use Phase Change Materials (PCMs) as heat storage media. Suitable PCMs have a temperature range of melting and solidification in accordance with a proposed application, a high heat of fusion, reliability, safety of use, and low cost.

This study investigates the PCMs that are suitable for integration in the interior of the building structures and have a melting range 22 and 28 °C. They are used in order to improve the thermal energy storage capacity (thermal inertia) of structures in different climate zones with different means of activation (passive or active).

The fact that PCMs transform from a solid to liquid state during the operation necessitates appropriate encapsulation. One option is the so-called macroencapsulation. It represents a common method of mechanical packaging [7] where the PCM is filled into macro-capsules (containers) of a required shape and size. The geometry of the container, its material composition, and thickness of walls all depend on the intended purpose of the particular LHTES system. The design of these parameters is crucial for flawless heat exchange between the PCM and the surrounding environment, as well as the reliability and durability of the system. There are three major container-related issues affecting the reliability of the LHTES [8,9,10]: (a) tightness of the container as the migration of PCM or components can lead to loss of heat storage capacity or undesirable interaction with the indoor environment, (b) stability of the container in contact with PCM, e.g., corrosion of metals due to exposition to PCM, (c) elimination of chemical reaction between PCM and container, i.e., the material of the container must be chemically compatible with PCMs.

These issues suggest that the compatibility of the heat storage media with the container material is a key parameter for ensuring the long service life of LHTES. Moreover, containers must have the necessary strength and flexibility to withstand the stresses caused by the volume changes during the phase change. Container materials also have to be UV stable and allow the rapid heat transfer for charging and discharging of PCMs. Metals are, therefore, often suggested as container materials, especially for the PCMs with a high melting range, which excludes other solutions (e.g., plastics). The advantages of metals include high thermal conductivity and regular crystalline structure serving as a barrier for diffusion of small gas molecules. On the other hand, they are susceptible to corrosion. Three types of PCM-induced corrosion of metals can occur [10]: (a) oxidation of the metal—PCM corrupts the porous surface layer of the container, which leads to its uniform perforation (typical for mild steel), (b) pitting corrosion—corrosion starts at a point, accelerates and produces deep “pits” (typical for stainless steel, aluminum, and coated metals with pinholes), (c) stress crack corrosion—corrosion occurs at stress region and results in sudden failure (typical for stainless steel).

There are already numerous studies evaluating the compatibility of PCMs with container materials [11]. Most of the studies focus on the compatibility between organic and inorganic PCMs with carbon steel, aluminum, copper, brass, and stainless steel. The corrosion resistance of metals exposed to PCMs in low-temperature LHTES is commonly assessed by calculated corrosion rates (CR) in these studies [12]. The procedure for the preparation of metal samples, for corrosion removal and evaluation of the corrosion damage, is standardized in ASTM G1 [13].

**Figure 1:**
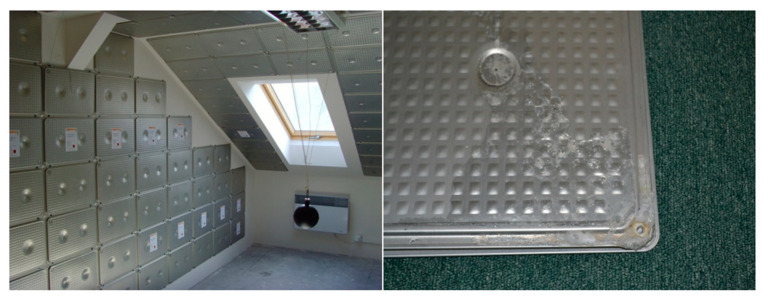
Application of PCM encapsulated in aluminum container: (**left**) Experimental installation in the test room at the Faculty of Civil Engineering, Brno University of Technology; (**right**) PCM leakage on the surface of aluminum container with salt hydrate PCMs placed on the wall of the test room for three years.

This issue of metal-PCM compatibility is stressed by the authors of this paper, who performed a three-year experiment focused on the influence of PCMs on the indoor environment [14,15]. Figure 1 (left) shows the installation of aluminum containers fixed on the walls and suspended ceiling in the test room at Brno University of Technology. The original aim of this installation was to evaluate the suitability of the LHTES integration into building structures for improving indoor thermal comfort. However, leakage of PCM from many containers (Figure 1, right) was observed during and after the experiment. This was the starting point of the authors’ research on PCM-metal compatibility. The present study follows the literature review below. The aim of the study is to evaluate the impact of long-term exposure to PCMs on selected metals: aluminum, brass, copper, and carbon steel using the CR calculation. Moreover, the visual changes were recorded to illustrate the effect of the PCMs on the surface of metals.

### 1.1. Literature Review on Compatibility Tests

Many existing studies evaluate the compatibility of metals and inorganic PCMs. The assessment of the compatibility between PCM (magnesium nitrate—PlusIce S83) and three metals (aluminum alloy, copper alloy, and carbon steel) was presented by Calabrese et al. [16]. They performed short-time immersion corrosion tests at 120 °C and applied two electrochemical methods for the evolution of metal degradation during immersion in PCM—open circuit potential measuring and electrochemical impedance spectroscopy. The electrochemical activity of metals immersed in PCM was analyzed by the evolution of the impedance curves at increasing immersion time. The results of the study indicate that carbon steel and copper alloy are unsuitable container materials due to their low electrochemical stability. The aluminum alloy was evaluated as a suitable container material for the selected PCM.

Farrell et al. [17] examined the corrosion resistance of aluminum alloys and copper in contact with commercially available salt hydrate PCMs PlusICE E17 and ClimSel C18. Firstly, they tested separate samples of each metal immersed in the PCMs. The results of this test showed a higher mass loss of copper samples. Secondly, they tested galvanic corrosion of combined samples of aluminum alloy and copper immersed in the PCMs. The authors concluded that aluminum fins combined with copper heat pipes in a heat exchanger are prone to galvanic corrosion caused by both examined PCMs.

Cabeza et al. [18] evaluated the behavior of five metals (aluminum, brass, copper, steel, and stainless steel) immersed in selected salt hydrate PCMs (zinc nitrate hexahydrate, sodium hydrogen phosphate dodecahydrate, and calcium chloride hexahydrate). The melting temperatures of the tested PCMs were 32 °C and 36 °C. The evaluation was based on the calculation of CRs and mass loss per meter and day. The samples were removed from the PCMs after three, seven, and 14 days. The results suggest that aluminum and steel should be avoided in PCM containers due to their high CRs. Slower corrosion was observed in the case of brass and copper. Stainless steel was evaluated as corrosion resistant.

The immersion corrosion test with the same metals and two salt hydrates (sodium acetate trihydrate, and sodium thiosulfate pentahydrate) with melting temperatures of 58 and 48 °C, respectively, was presented by Cabeza et al. [19]. In this case, the metal samples were immersed in pure PCMs and PCMs enhanced with graphite (for increased heat transfer). The immersion time was one, two, four, and ten weeks. Based on the results, the authors discouraged the application of brass and copper in containers for long-term storage of the tested PCMs. Aluminum, steel, and stainless steel proved to be suitable container materials for both PCMs; however, the authors recommended monitoring of steel in contact with graphite.

The study by Fernández et al. [20] generally recommended stainless steel as a container material for inorganic PCMs. They evaluated CRs and industry recommendations of six metals (aluminum, brass, copper, steel, stainless steel, and carbon steel) exposed to 13 salt hydrate PCMs. The study concluded that carbon steel is not recommended or cautiously recommended for the tested PCMs.

Ushak et al. [21] investigated the corrosion effect of bischofite (natural salt composed primarily of MgCl_2_ × 6H_2_O) and commercial MgCl_2_ × 6H_2_O (more than 99% purity) on copper, aluminum and stainless steel. The exposure times were 250, 500, 750, and 1000 h. The calculated mass loss was used as the criterion for the evaluation of the corrosion effect. The highest mass loss was calculated for copper, while stainless steel has shown the lowest mass loss.

Moreno et al. [22] tested corrosion potential of five inorganic PCMs for cooling applications (melting range 10–15 °C) and six inorganic PCMs for the heating application (melting range 45–48.5 °C). They selected copper, aluminum, and stainless steel as potential container materials. The samples of these metals were immersed in test tubes containing PCMs at the constant temperature 22 °C (cooling application) or 60 °C (heating application) for one, four, and 12 weeks. The results confirmed the corrosion resistance of stainless steel in all tested inorganic PCMs stated in previous studies.

The same result was achieved in immersion tests performed by Oró et al. [23]. They also tested copper, aluminum, and carbon steel. Additionally, they found that corrosion was significantly slowed down by adding 1% of oxyethylmethylcellulose to thicken the solution in their tests.

Danielik et al. [24,25] investigated the corrosion resistance of carbon steel and copper immersed in four PCMs: Mg(NO_3_)_2_ × 6H_2_O, Mg(NO_3_)_2_ × 6H_2_O + 0.5wt% Mg(OH)_2_, Mg(NO_3_)_2_ × 6H_2_O + 0.5wt% Sr(OH)_2_, Mg(NO_3_)_2_ × 6H_2_O + Ca(NO_3_)_2_ × 4H_2_O (1:1), and Ca(NO_3_)_2_ × 4H_2_O. Melting temperatures of the tested PCMs were 42.7–88.9 °C. Magnesium hydroxide and strontium hexahydrate were added due to the stabilization of the supercooling of magnesium nitrate hexahydrate. The ambient temperature was repeatedly changed from 20 °C up to a temperature slightly above the melting range of each PCM during immersion. The comparison of results in both papers [24,25] shows that the CR of carbon steel (4.6–33.3 mg·cm^−2^·year^−1^) is much higher than the CR of copper (1.5–8.9 mg·cm^−2^·year^−1^).

Zhao et al. [26] studied inorganic hydrated PCM composite Na_2_HPO_4_ × 12H_2_O − Na_2_SO_4_ × 10H_2_O with the composition ratio of 9:1 as an efficient LHTES medium. The pH of PCM was modified by H_2_SO_4_ and NaOH to prepare five different pH values. Aluminum Al-1060 sheets served as working electrodes. The corrosion property was investigated electrochemically (DC polarization). The microstructural characterization by optical microscope and analysis by scanning electron microscope were applied for the evaluation of the experiments. The results showed that the pH of PCM highly influenced the corrosion potential.

Dorcheh et al. [27] published corrosion tests at 600 °C for two low chromium alloys (P91 and X20CrMoV), two high chromium stainless steel (SS347H and SS316), and Ni-alloy (IN625) immersed for a long time in molten eutectic composition (40% KNO_3_ and 60% NaNO_3_) for up to 5000 h. The evaluation was based on the macroscopic observations and metallographic investigation. The authors proved that IN 625 showed the best corrosion performance. On the other hand, low chromium steels have poor long-term resistance. Due to the high price of Ni-alloys, the authors recommend the use of low alloyed steel with an additional protective layer.

Many published studies also focused on organic PCMs and their corrosion potential. Kahvaji et al. [28] selected 16 samples of potential container materials or supplementary gasket materials. These included nine metals or metal alloys and seven plastics. The study focused on the compatibility potential of these materials and six organic PCMs (five fatty acids and one alcohol). Materials were immersed in liquid PCMs with a temperature of 75 °C for one, six, and 12 weeks. The evaluation was based on the results of the CR calculations. The results showed that only stainless-steel alloys and aluminum alloys were compatible with all PCMs.

Ferrer et al. [29] tested the compatibility of selected metals (stainless steel SS-316 and SS-304, copper, aluminum, and carbon steel), two commercially available PCMs (inorganic SP21E and bio-based PureTemp 23) and two fatty acid eutectics. The melting range of all four PCMs was between 21 °C and 23 °C. The metals were immersed in PCMs for one, four, and 12 weeks. Based on the CR calculations, the results of the study concluded that all the tested metals could be recommended as containers for bio-based PCM (PureTemp 23). Both fatty acid eutectics caused only low corrosion of copper samples, so the tested metals could be used in their containers too. Stainless steel and copper also proved resistant to the inorganic PCM.

Sari et Kaygusuz [30] presented a study focused on the thermal stability of stearic acid, palmitic acid, myristic acid, and lauric acid and their compatibility with carbon steel, stainless steel, aluminum, and copper. The evaluation was based on gravimetric and metallographic analysis. Highest CRs were calculated for carbon steel and copper in contact with myristic acid and copper immersed in stearic acid. In contrast, the lowest CR was calculated for stainless steel.

Browne et al. [31] chose aluminum, brass, copper, stainless steel, and mild steel for corrosion experiments with capric acid, capric-palmitic acid, capric-lauric acid, sal hydrate SP22, and Micronal^®^. The authors concluded that only stainless steel was a suitable container material for all the tested PCMs. All metals except aluminum were recommended for use in combination with SP22. Similarly, all metals except mild steel were recommended for the applications with Micronal^®^. Caution was recommended for other metal-PCM pairs.

Dheep et Sreekumar [32] investigated the corrosion potential of organic PCM phenylacetic acid (PAA) for its proposed integration as an LHTES unit in the solar air heater. Aluminum, copper, and stainless steel SS 304 were chosen for the compatibility tests with PAA. The corrosive nature of PAA was evaluated by mass loss and corrosion rate. The mass losses were 9.09% for copper, 3.20% for aluminum, and 3.43% for stainless steel. The highest corrosion rate of 11.9381 mg·cm^−2^·a^−1^ was calculated for copper.

The compatibility of glutaric acid as an organic PCM with aluminum, copper, and stainless steel as containment material was tested by Dheep and Sreekumar [33] as well. The results correspond with study [32], and show that the corrosion rate of copper was 10.9869 mg·cm^−2^·a^−1^, which was much higher when compared to the corrosion rate of 0.3004 mg·cm^−2^·a^−1^ for aluminum.

The literature review summarized in Table 1 has shown the necessity for further compatibility tests of metals (containers) and PCMs. It also shows a lack of knowledge on the performance of commercially available PCMs (in metal containers). The purpose of this study is to formulate recommendations for the compatibility of the tested metals and the selected organic and inorganic PCMs. To our knowledge, no relevant study has described the compatibility of the metals and PCMs selected for this study.

## 2. Results

This section presents the results of the compatibility tests described in Section 4. The evaluation of the results is divided in two parts. The first part focuses on the visual evaluation of the samples, i.e., the color, shape, and other changes on the surface of samples encountered after their removal from the PCMs. The second part presents the evaluation of the CRs that were calculated according to Equation (1).

**Figure 2:**
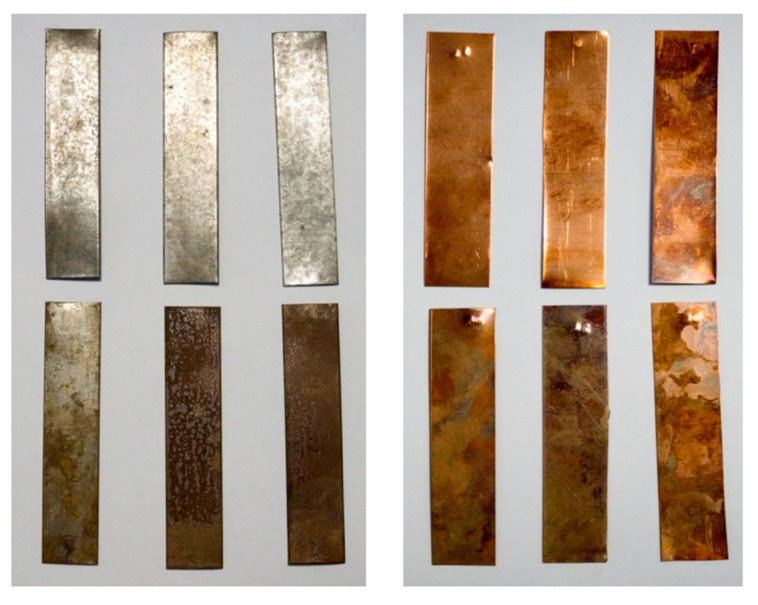
Corrosion, change of appearance, and color of the metal samples after testing. On the left side, carbon steel samples removed from organic PCM Linpar 1820 (top row) and inorganic PCM Rubitherm RT SP25 (bottom row) after one, four, and twelve weeks (left to right). On the right-side, copper samples removed from organic PCM Linpar 17 (top row) and inorganic PCM Rubitherm RT SP25 (bottom row) after one, four, and twelve weeks (left to right).

Immersion in organic PCMs caused no visual changes on the surface of brass, aluminum, and carbon steel samples. Local surface changes (darker stains) and tarnishing occurred on the copper samples. The extent of these changes increased with longer exposure to the PCMs (see Figure 2, top). More pronounced changes were encountered on the samples immersed in the inorganic PCMs. All samples were tarnished as early as one week after immersion. Noticeable dark brown stains appeared on the surface of the copper and brass samples. Corrosion also occurred on the surface of the copper (blue corrosion) and carbon steel (dark red and brown corrosion) samples. The progress of tarnishing and corrosion in time is visible in Figure 2 (bottom).

**Figure 3:**
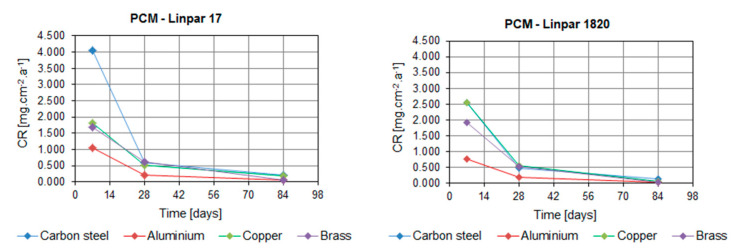
Dependence of corrosion rate of the tested metals on the time of immersion in organic PCMs: (**left**) Linpar 17; (**right**) Linpar 1820.

**Figure 4:**
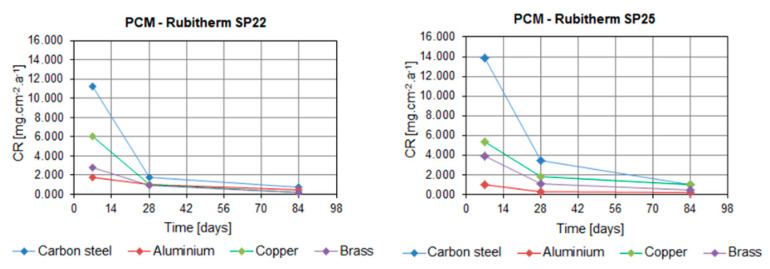
Dependence of the CR of tested metals on the time of immersion in inorganic PCMs: (**left**) in Rubitherm SP22; (**right**) in Rubitherm SP25.

**Figure 5:**
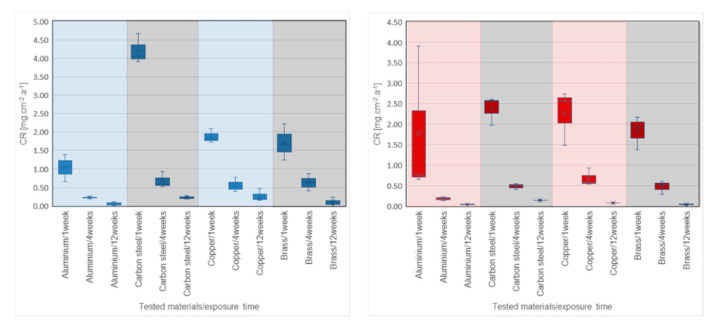
Box charts with CRs of metals immersed in organic PCMs: (**left**) in Linpar 17; (**right**) in Linpar 1820. The charts show the distribution of the calculated data based on the statistical deviations for each metal and sample set after one, four, and twelve weeks (left to right) immersion time.

**Figure 6:**
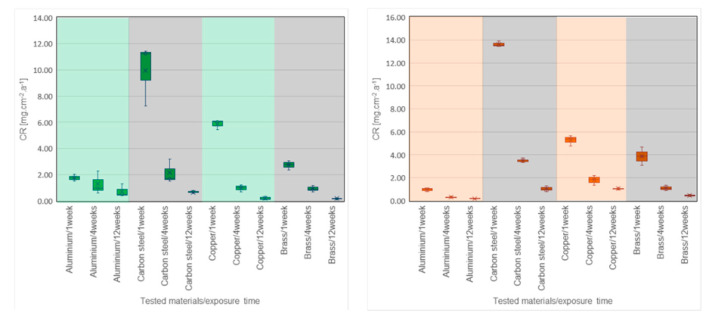
Box charts with CRs of metals immersed in inorganic PCMs: (**left**) in Rubitherm SP22; (**right**) in Rubitherm SP25. The charts show the distribution of the calculated data based on the statistical deviations for each metal and the samples set after one, four, and twelve weeks (left to right) immersion time.

Table 2, Figure 3 and Figure 4 show the CR values of the tested metals in all PCMs. The distribution of data based on the determination of statistical deviations is shown in Figure 5 and Figure 6. Median, 1st, and 3rd quartile, minimum and maximum values were calculated for each sample set (three pieces of a particular metal). Higher dispersion of results occurred in sample sets immersed in organic PCMs, especially Linpar 1820. The widest dispersion was calculated in the case of aluminum, which also suffered the worst surface corrosion.

**Figure 7:**
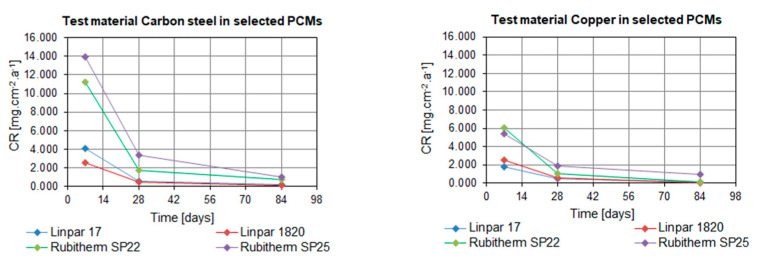
Comparison of the dependence of CR of selected metals in both types of organic and inorganic PCMs: (**left**) carbon steel samples; (**right**) copper samples.

When considering the organic PCMs, the results show that higher CRs were achieved with the long-term immersion of the samples in Linpar 17. The highest values were achieved in case of the carbon steel, where the CR exceeded 4.0 mg·cm^−2^·year^−1^. It should be noted that the copper and brass samples immersed in Linpar 1820 had higher CR after one-week immersion, and then it slowed down. The difference was almost 30% in the case of copper. The CR curves of metal samples immersed in inorganic PCMs (Figure 4) are very similar to those in organic PCMs. The difference is in their scale: the CRs of metals in inorganic PCMs are much higher. This is illustrated in Figure 7, which shows CR curves of copper and carbon steel (highest CRs of all four metals). The values gradually increase from aluminum to brass, copper, and finally carbon steel. The highest values of CR were calculated for carbon steel in Rubitherm SP25 (up to 13.897 mg·cm^−2^·year^−1^) and copper in Rubitherm SP22 (up to 6.049 mg·cm^−2^·year^−1^).

Another evaluated parameter was the mass loss Δ*m*, which is an important factor for the evaluation of the corrosion effect (see Section 4 for details). The highest mass loss was calculated for copper and carbon steel samples in Rubitherm SP25. It reached 9.6 mg for copper after 12 weeks of immersion and 11.2 mg for carbon steel after four weeks of immersion. In contrast, all metals achieved the lowest mass losses when immersed in organic PCM Linpar 17. The mass loss of the aluminum samples immersed in this PCM ranged between 0.5 to 0.8 mg, depending on the length of the immersion. This is several times lower compared to aluminum samples in inorganic PCMs Rubitherm SP22 (Δ*m* 1.4 to 4.4 mg).

## 3. Discussion

The results of the study are compiled in Table 3. It shows the range of calculated CR values, surface changes, the occurrence of corrosion, and the evaluation according to the guide for corrosion weight loss used in the industry [23,29]. Most of the tested metal-PCM combinations were evaluated as “Recommended” according to the guide. The exception is carbon steel in combination with inorganic PCMs, which achieved “Cautiously recommended” levels. Regardless of the guide, it seemed appropriate to discourage further use of copper and carbon steel in combination with inorganic PCMs due to higher CR values, significant mass losses, and visible changes on the surface of the samples.

The results of the study also confirm the literature review (see Section 1.1) regarding significant differences in the suitability of metals exposed to organic and inorganic PCMs (in favor of organic PCMs). All metals demonstrated good compatibility with organic PCMs—minimum to no visual changes, lower CRs, and mass losses where the maximum mass loss of metal in the organic PCM was 2.1 mg in the case of carbon steel exposed to Linpar 1820. This was almost six times lower in value compared to the mass loss of carbon steel in the inorganic PCM Rubitherm SP25. The results indicate low aggressiveness of tested organic PCMs and confirm suitability of tested metals for container use.

This makes organic PCMs readily available for suggested applications in buildings. Interestingly enough, there is a lack of literature regarding the performance of organic PCMs and their applications in LHTES. In contrast, cited studies often focus on corrosive effects of inorganic PCMs such as MgCl_2_ × 6H_2_O [21], Zn(NO_3_)_2_ × 6H_2_O, Na_2_HPO_4_ × 12H_2_O, or CaCl_2_ × 6H_2_O [18], which cannot be utilized in building structures in their pure form. It should be highlighted that the results presented in this study are more accurate than the results of studies cited in the literature review (e.g., [18,19,22,23]). This is due to the fact that CR is calculated using statistically processed results (median of weight loss) of three samples of each metal at each measurement. In contrast, cited studies often base their calculations on the measurements of individual samples. Another advantage of the presented study is the fact that it evaluates PCMs that are commonly available at the market. Stainless steel tested in many studies [18,20,31,33] was not selected for our compatibility test because it is generally recommended for use as container or heat exchanger material.

## 4. Materials and Methods

### 4.1. Materials

Long-term experimental measurements were performed to verify the compatibility of two types of PCMs (organic and inorganic) with selected metals (representing possible container materials) in this study. The aim of the experiment was to identify the metals suitable for the role of long-term PCM containers (integrated into building structures).

**Figure 8:**
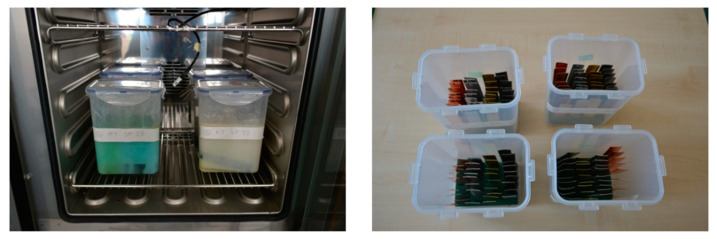
Metal samples prepared for corrosion testing: (**left**) Test beakers placed in incubator; (**right**) Samples in test beakers before the test.

Based on the literature review in Section 1, four metals applicable in the role of PCM containers (Figure 8) were selected for the experiment—product name (according to standard): carbon steel DC01-A-m (EN 10 130), aluminum AW 1050 H111 (DIN 3,0255), copper CW024A (EN 1652), and brass CW508L (EN 1652). Stainless steel was also considered; however, the literature review has shown sufficient data regarding its compatibility with both organic and inorganic PCMs. The reasons for the selection of the particular metals are low costs and worldwide availability. Thirty-six samples were prepared out of each metal (144 samples in total) for the testing (see Figure 10, right). The samples of each metal were divided into 12 sets of three. Individual samples were approximately 2.0 cm wide, 10.0 cm high, and 0.1 cm (carbon steel) or 0.05 cm (other metals) thick.

Table 4 lists tested PCMs and their (selected) parameters. Paraffin-based Linpar 17 and Linpar 1820 (Sasol Germany GmbH, Hamburg, Germany) represent organic PCMs in the experiment, while Rubitherm SP22 and Rubitherm SP25 (Rubitherm Technologies GmbH, Berlin, Germany) represent inorganic PCMs based on saltwater mixtures and additives (with neutral pH). The table also specifies the selected parameters of the PCMs acquired using Different Scanning Calorimeter (DSC). Detailed study based on the DSC and TGA analysis of commercial PCMs was published in the past [34]. Table 4 shows that all selected PCMs have peak temperatures between 22 and 28 °C. This suggests the suitability of the particular PCMs for integration inside buildings as these temperatures are similar to indoor temperatures in most (residential and office) buildings. Thus, the application of the PCMs could increase the heat storage capacity of interior building structures and help to maintain a suitable indoor environment. All tested PCMs are available at the European market and could be integrated into building structures and fulfill requirement for use in buildings. Contrary to studies presented in the past [18,21,24,25], selected PCMs are ready to use in building interior without additional adjustments.

### 4.2. Experimental

**Figure 9:**
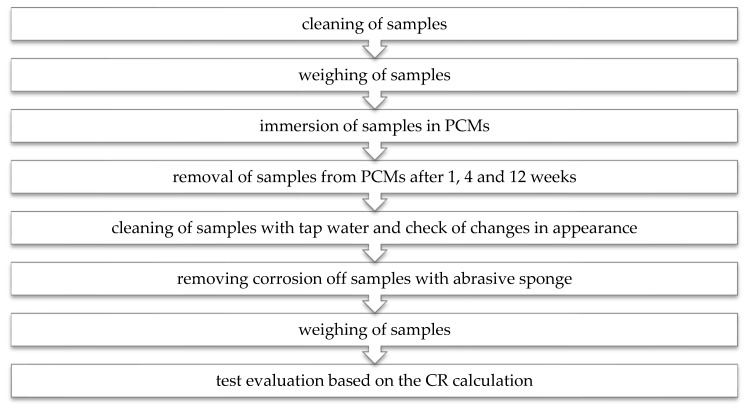
Experimental procedure.

The evaluation was based on monitoring of weight loss, and visual surface changes (i.e., corrosion, change of color, swelling of the samples) of the selected metal samples. To assess the compatibility, the metals were physically tested by immersion tests to establish their CR based on calculated mass loss. The procedure of physical testing is summarized in Figure 9.

The main parameter evaluating the compatibility of metal-PCM combinations in this study is the corrosion rate (CR) defined according to the methodology described in [23] and [29]. The international guide for corrosion weight loss used in the industry [22,23,29] (see Table 5) was utilized for the classification and verbal evaluation of CR in this study. CR was defined as a change of mass ∆*m* relative to the surface area of the metal sample in a specific experimental time period. The change in mass was defined as the difference in the initial sample weight and the weight at the end of the particular time period.

*CR* = ∆*m*/*A* (*t_0_* − *t*), (1)

where *CR* was the corrosion rate in mg·cm^−2^·year^−1^, ∆*m* was mass loss (in mg), and it was defined by the relation ∆*m* = *m*(*t_0_*) − *m*(*t*). The value *m*(*t_0_*) was the initial weight of the sample (in mg) before immersion in PCMs, and *m*(*t*) was the final weight of the sample (in mg) after removal from PCMs. The surface area of each sample was expressed by value A (in cm^2^), and (*t_0_* − *t*) was the experimental time of PCMs exposure (in years).

**Figure 10:**
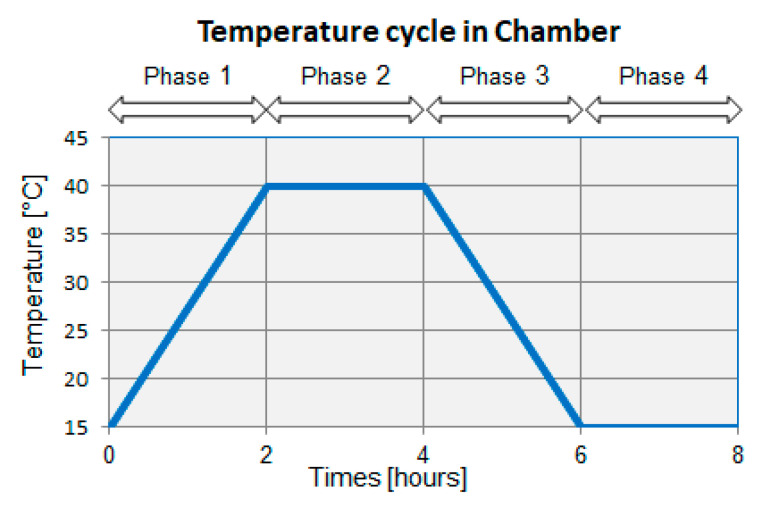
Temperature cycle repeated in the Peltier-cooled incubator for the duration of the experiment.

All metal samples were clearly labeled, documented, and the excessive material (residuals after cutting) was mechanically removed. After that, the samples were purified by water and degreaser, dried and weighed with the analytical balance Radwag AS220.R2 (RADWAG, Radom, Poland). This balance meets OIML class 1 requirements with an accuracy of 0.1 mg. Afterward, the samples were visually checked, inserted into supporting foam holder (see Figure 10, right), and placed into testing beakers. There they were fully immersed in liquid PCMs. Special care was taken to prevent mutual contact of the metal samples (causing uneven exposure of the surface to the PCM).

The test beakers were placed into Peltier-cooled incubator Memmert IP 55 PLUS (Memmert GmBH + Co. KG, Büchenbach, Germany) (see in Figure 10, left). There they were exposed to repeating temperature cycling with temperatures ranging from 15 °C to 40 °C. The cycle consisted of four phases, each lasting for two hours (see Figure 10). The cycle started with linear temperature rise (to 40 °C) during the first phase. The second phase comprised maintaining the temperature at this level. The temperature was linearly reduced to 15 °C in the third phase. This temperature was maintained during the fourth phase of the cycle. The temperature cycling lasted for twelve weeks (84 days).

Individual sets of samples were withdrawn from the beakers at the given time after one week (seven days), four weeks (28 days), and 12 weeks (84 days) of exposure to the PCMs.

After the removal from the test beakers, the samples were visually inspected, and the level of corrosion/changes was recorded (i.e., photographed)—see Figure 2. Then the samples were cleaned of any liquid residues with water, and any surface corrosion was removed using an abrasive sponge. Finally, the samples were dried with a cloth. These cleaned samples were weighed on the analytical balance. The weight of each sample was rounded to 0.1 mg.

One sample from each three pieces of each metal set was selected for the subsequent calculations using the median value. The weight change of the selected samples was used for calculating CR afterward. This method was selected to minimize errors and eliminate extreme values encountered in the literature review.

## 5. Conclusions

This study explores the compatibility of selected metals and (organic and inorganic) PCMs in order to select suitable metals for the role of container material for long term PCM encapsulation in building interiors. The samples of four metals (aluminum, brass, copper, and carbon steel) were tested in combination with four PCMs for up to 12 weeks to find the most suitable metal-PCM combination.

The initial premise of the experiment was that the exposure of the surface of the metals to the effects of PCMs would affect their shape, surface flatness, color, and perhaps the occurrence of surface corrosion will be detected. The literature review suggested that copper and carbon steel are unsuitable for long-term exposition to PCMs. This suggestion was confirmed as both metals have shown signs of surface corrosion and change of color at the end of the experiment. Moreover, they both achieved higher CRs and more pronounced mass loss compared to other tested metals (in inorganic PCMs). The results indicate that aluminum is the best (out of the tested metals) suited for the role of PCM container in building interior. It had the lowest mass loss and CR, and it suffered only minimal visual changes during the experiment. This long-term stability should ensure the durability of the whole LHTES system and prevent leakage due to corrosion.

To summarize the main points, the research has revealed several facts about the compatibility of the selected PCMs and metals.

Calculation of the CR based on the median of weight loss (three samples of each metal at each measurement) brings more reliable results in comparison to the reviewed studies.

Long-time duration of immersion of metals in PCMs is the second advantage of the presented study. The problem with the classification of corrosion weight loss was detected because the coarse-scale (see Table 5) of CR is not so suitable for the evaluation of metal-PCMs pairs. Therefore, to create a finer scale with lower CR values is now a challenge not only for the authors of the study.

The results of this study are important, especially when the commercially available selected PCMs are intended for integration in the interior of buildings. The long-term testing of modules containing organic-based PCMs, which will be performed under real boundary conditions will follow in the near future. Therefore, the results from our tests have higher importance for follow-up full-scale experiments compared to other studies testing inorganic PCMs (salts hydrates), which are not ready to use in building interior.

## Figures and Tables

**Table 1:** Summary of the selected studies on corrosion on metal containers in contact with PCMs.

Authors	Subject of Investigation	Main Results
Browne et al. [31]	Compatibility tests of aluminum, brass, copper, stainless steel and mild steel in contact with capric acid, capric-palmitic acid, capric-lauric acid, salt hydrate SP22, and Micronal^®^.	Stainless steel is suitable container material for all tested PCMs.
Cabeza et al. [18]	Tests of aluminum, brass, copper, steel and stainless steel in contact with Zn(NO_3_)_2_ × 6H_2_O, Na_2_HPO_4_ × 12H_2_O and CaCl_2_ × 6H_2_O	Aluminum and steel should be avoided in PCM containers due to their high corrosion rates; slower corrosion was observed for brass and copper. Stainless steel is corrosion resistant.
Cabeza et al. [19]	Tests of aluminum, brass, copper, steel, and stainless steel in contact with NaOAc × 3H_2_O and Na_2_S_2_O_3_ × 5H_2_O (in pure form and enhanced with graphite).	Aluminum, steel, and stainless steel are suitable container materials for the tested PCMs, although authors recommended monitoring of steel in contact with graphite.
Calabrese et al. [16]	Compatibility between magnesium nitrate PlusIce S83 and aluminum alloy, copper alloy, and carbon steel	Aluminum alloy is suitable container material for Plus Ice S83 PCM. Carbon steel and copper alloy suffer too low electrochemical stability.
Danielik et al. [24,25]	Corrosion resistance of carbon steel and copper in contact with Mg(NO_3_)_2_ × 6H_2_O, Mg(NO_3_)_2_ × 6H_2_O + 0.5 wt% Mg(OH)_2_, Mg(NO_3_)_2_ × 6H_2_O + 0.5 wt% Sr(OH)_2_, Mg(NO_3_)_2_ × 6H_2_O + Ca(NO_3_)_2_ × 4H_2_O (1:1) and Ca(NO_3_)_2_ × 4H_2_O.	Corrosion rate of carbon steel is much higher than corrosion rate of copper when exposed to tested PCMs.
Dheep and Sreekumar [32]	Corrosion analysis of phenyl acetic acid in contact with copper, aluminum, and stainless steel.	Phenyl acetic acid is less corrosive and compatible with stainless steel. The higher corrosion rate was calculated for copper.
Dheep and Sreekumar [33]	Compatibility tests for glutaric acid and copper, aluminum, and stainless steel	The lowest corrosion rate and the lowest mass loss were calculated for stainless steel
Dorcheh et al. [27]	Tests for two low chromium alloys, two high chromium stainless steels, and Ni-alloy immersed in molten eutectic composition.	The best material is Ni-alloy, alloyed steels are recommended with additional protective coatings.
Farrell et al. [17]	Corrosion resistance of aluminum alloy and copper in contact with PCMs PlusICE E17 and ClimSel C18.	Aluminum combined with copper in a heat exchanger is prone to galvanic corrosion due to exposure to tested PCMs.
Fernández et al. [20]	Compatibility tests of aluminum, brass, copper, steel, stainless steel and carbon steel in contact with 13 salt hydrates.	Carbon steel is not recommended or cautiously recommended as a container material for the tested PCMs. Stainless steel is recommended as container material for inorganic PCMs
Ferrer et al. [29]	Compatibility tests of stainless-steel SS-316 and SS-304, copper, aluminum, and carbon steel in contact with inorganic SP21E, bio-based PureTemp 23, and two fatty acid eutectics.	All tested metals are recommended as container materials for bio-based PCM. Stainless steel and copper also proved resistant to inorganic PCM.
Kahvaji et al. [28]	Study focused on the compatibility potential of 16 samples of container materials or supplementary gasket materials with five fatty acids and one alcohol.	Stainless steel alloys and aluminum alloys have shown good compatibility with all PCMs.
Moreno et al. [22]	Corrosion potential of five inorganic PCMs for cooling applications and six inorganic PCMs for heating application on copper, aluminum, stainless steel	Confirmed corrosive resistance of stainless steel exposed to the tested inorganic PCMs.
Oró et al. [23]	Study on corrosion of copper, aluminum, stainless steel carbon steel exposed to three commercial PCMs and seven PCMs with special formulation based on the NH_4_Cl + H_2_O	Only stainless steel is recommended as PCM container material. Copper and carbon steel should be avoided, and aluminum is not recommended for long term PCM storage.
Sari et Kaygusuz [30]	Study on compatibility of stearic acid, palmitic acid, myristic acid and lauric acid with carbon steel, stainless steel, aluminum, and copper.	The highest corrosion rates were calculated for carbon steel and copper in contact with myristic acid, the lowest corrosion rate was calculated for stainless steel.
Ushak et al. [21]	Corrosion effect of bischofite and commercial MgCl_2_ × 6H_2_O on copper, aluminum, and stainless steel.	Copper has the highest and stainless steel the lowest mass loss.
Zhao et al. [26]	Study on corrosion of aluminum Al-1060 immersed in molten hydrated salt PCM composite Na_2_HPO_4_ × 12H_2_O − Na_2_SO_4_ × 10H_2_O	Corrosion property of composite PCM is highly related with the pH.

**Table 2:** CRs of tested metals exposed to particular PCMs (organic Linpar and inorganic Rubitherm).

Metal	Exposure Time	Corrosion Rate (mg·cm^−2^·year^−1^)			
		Linpar 17	Linpar 1820	RT SP22	RT SP25
Carbon steel	1 week	4.058	2.562	11.236	13.897
	4 weeks	0.589	0.492	1.723	3.413
	12 weeks	0.214	0.135	0.712	1.029
Aluminum	1 week	1.049	0.762	1.765	1.024
	4 weeks	0.219	0.190	0.991	0.292
	12 weeks	0.053	0.033	0.460	0.194
Copper	1 week	1.807	2.564	6.049	5.400
	4 weeks	0.525	0.572	1.013	1.863
	12 weeks	0.174	0.072	0.152	1.004
Brass	1 week	1.685	1.926	2.795	3.882
	4 weeks	0.621	0.527	0.894	1.061
	12 weeks	0.044	0.040	0.168	0.476

**Table 3:** Classification of selected PCM–metal combinations according to the CR parameter [mg·cm^−2^·year^−1^] and recommendation according to the final appearance of samples (based on Table 5).

Material (Metals)	Criterion	PCMs (Organic Group Linpar and Inorganic Group RT)			
		Linpar 17	Linpar 1820	RT SP22	RT SP25
carbon steel	CR	0.21 to 4.05	0.13 to 2.56	0.71 to 11.23	1.02 to 13.89
	corrosion	no corrosion surface w/o change	no corrosion surface w/o change	corrosion–red color	corrosion–red color
	recommendation	recommended	recommended	cautiously recommended	cautiously recommended
aluminium	CR	0.05 to 1.05	0.03 to 0.76	0.46 to 1.76	0.19 to 1.02
	corrosion	no corrosion, shiny surface	no corrosion, shiny surface	no corrosion, surface tarnish, occurrence of cracks	no corrosion, surface tarnish
	recommendation	recommended	recommended	recommended	recommended
copper	CR	0.17 to 1.80	0.07 to 2.56	0.15 to 2.79	1.00 to 5.40
	corrosion	no corrosion, shiny to matt surface with time	no corrosion, shiny to matt surface with time	corrosion–blue color, surface tarnish	corrosion–blue color, surface tarnish
	recommendation	recommended	recommended	recommended	recommended
brass	CR	0.04 to 1.68	0.04 to 1.92	0.16 to 6.04	0.47 to 3.88
	corrosion	no corrosion, shiny surface	no corrosion, shiny surface	no corrosion, surface tarnish	no corrosion, surface tarnish
	recommendation	recommended	recommended	recommended	recommended

**Table 4:** Parameters of selected PCMs with DSC.

Type	Product Name	Manufacturer	Latent Heat [J∙g^−1^]	Onset Temperature [°C]	Peak Temperature [°C]
inorganic	SP22	Rubitherm Technologies GmbH, Berlin, Germany	145	14	25
inorganic	SP25	Rubitherm Technologies GmbH, Berlin, Germany	122	18	28
organic	Linpar 17	Sasol Germany GmbH, Hamburg, Germany	152	21	22
organic	Linpar 1820	Sasol Germany GmbH, Hamburg, Germany	141	24	27

**Table 5:** Guide for corrosion weight loss used in the industry [23,29].

CR [mg·cm^−2^·year^−1^]	Recommendation
>1000	Completely destroyed within days
100–999	Not recommended for service greater than a month
50–99	Not recommended for service greater than one year
10–49	Caution recommended, based on the specific application
0.3–9.9	Recommended for long term service
<0.2	Recommended for long term service; no corrosion, other than as a result of surface cleaning, was evidenced
